# Risk assessment of malaria in land border regions of China in the context of malaria elimination

**DOI:** 10.1186/s12936-016-1590-1

**Published:** 2016-11-08

**Authors:** Qian Zhang, Junling Sun, Zike Zhang, Qibin Geng, Shengjie Lai, Wenbiao Hu, Archie C. A. Clements, Zhongjie Li

**Affiliations:** 1 Division of Infectious Disease, Key Laboratory of Surveillance and Early-warning on Infectious Disease, Chinese Center for Disease Control and Prevention, 155 Changbai Rd, Changping District, Beijing, 102206 China; 2 Center of Clinical Laboratory, the First Affiliated Hospital, College of Medicine, Zhejiang University, Hangzhou, China; 3 State Key Laboratory of Virology and College of Life Sciences, Wuhan University, Wuhan, China; 4 School of Public Health and Social Work, Queensland University of Technology, Brisbane, Australia; 5 Research School of Population Health, College of Medicine, Biology and Environment, The Australian National University, Canberra, Australia

**Keywords:** Malaria, Border, Epidemiology, Elimination, China

## Abstract

**Background:**

Cross-border malaria transmission poses a challenge for countries to achieve and maintain malaria elimination. Because of a dramatic increase of cross-border population movement between China and 14 neighbouring countries, the malaria epidemic risk in China’s land border regions needs to be understood.

**Methods:**

In this study, individual case-based epidemiological data on malaria in the 136 counties of China with international land borders, from 2011 to 2014, were extracted from the National Infectious Disease Information System. The Plasmodium species, seasonality, spatiotemporal distribution and changing features of imported and indigenous cases were analysed using descriptive spatial and temporal methods.

**Results:**

A total of 1948 malaria cases were reported, with 1406 (72.2%) imported cases and 542 (27.8%) indigenous cases. *Plasmodium vivax* is the predominant species, with 1536 malaria cases occurrence (78.9%), following by *Plasmodium falciparum* (361 cases, 18.5%), and the others (51 cases, 2.6%). The magnitude and geographic distribution of malaria in land border counties shrunk sharply during the elimination period. Imported malaria cases were with a peak of 546 cases in 2011, decreasing yearly in the following years. The number of counties with imported cases decreased from 28 counties in 2011 to 26 counties in 2014. Indigenous malaria cases presented a markedly decreasing trend, with 319 indigenous cases in 2011 reducing to only 33 indigenous cases in 2014. The number of counties with indigenous cases reduced from 26 counties in 2011 to 10 counties in 2014. However, several bordering counties of Yunnan province adjacent to Myanmar reported indigenous malaria cases in the four consecutive years from 2011 to 2014.

**Conclusions:**

The scale and extent of malaria occurrence in the international land border counties of China decreased dramatically during the elimination period. However, several high-risk counties, especially along the China–Myanmar border, still face a persistent risk of malaria introduction and transmission. The study emphasizes the importance and urgency of cross-border cooperation between neighbouring countries to jointly face malaria threats to elimination goals.

## Background

Malaria is one of the most important parasitic infections in human beings. The condition is caused by infection by one or more of five *Plasmodium* species via the bite of infected female *Anopheles* mosquitoes [1, 2]. According to the World Health Organization (WHO) estimates, in 2015, 3.2 billion people were at risk of being infected with malaria and developing the disease, and 214 million cases of malaria and 438,000 deaths occurred globally [3]. As a result of efforts and progress made by the international community, global malaria incidence decreased dramatically between 2000 and 2010 [4]. In 2015, the WHO set the ambitious new target of reducing the global malaria burden by 90% by 2030, and it encouraged member states to fulfill the goal of malaria elimination [5]. However, many countries face challenges in achieving the elimination goal because of the threat of malaria importation and re-introduction [6], especially in border areas adjacent to high malaria endemic countries. In South Korea, for example, there had been no indigenous malaria cases after 1984, until the re-emergence of *P. vivax* in the demilitarized zone showed that malaria transmission along the border of North Korea would continue to be a challenge [7].

China has had remarkable success in controlling locally transmitted malaria through several initiatives facilitated by increased funding, including: effective vector control, strengthening of health systems, improving case management with more effective treatment regimens, and enhanced case reporting and surveillance [8]. Since 2010, annual numbers of reported malaria cases have fallen to unprecedentedly low levels, with only hundreds of autochthonous malaria cases now occurring in limited areas [9–11]. In 2010, the Chinese government launched the national malaria elimination programme with the goal of eliminating malaria nationwide by 2020 [12]. However, China shares more than 22,000 km of land border with 14 neighbouring countries, six of which are still malaria-endemic [3, 13, 14]. Malaria from these countries, especially Myanmar poses a major threat to the achievement and maintenance of national malaria elimination [15]. Although there were checks in international land borders, population movement between China and other malaria-endemic countries becoming even more frequent was still a high risk of malaria infection. The epidemiological situation in these high-risk areas needs to be further investigated. In this study, the changing risk of malaria occurrence in all counties with international land borders was explored following the initiation of the Chinese malaria elimination programme in 2010, in order to identify remaining high-risk areas, formulate response measures and allocate resources for malaria elimination.

## Methods

### National Malaria Surveillance Programme

Malaria cases are diagnosed according to the unified diagnostic criteria issued by the Chinese Ministry of Health, including clinically diagnosed and laboratory confirmed cases. All probable or laboratory confirmed cases are reported to the Chinese Center for Disease Control and Prevention (China CDC) in Beijing. The dataset used in this study consists of individual malaria cases reported by doctors within 24 h of diagnosis through the web-based National Notifiable Infectious Disease Reporting Information System at the China CDC from 2011 to 2014 [16, 17]. Health workers in both the public and private medical sectors were required to report malaria cases. The individual data include gender, age, address, nationality, date of illness onset, type of diagnosis, imported or indigenous status and laboratory test result. All the data used in this study were anonymized such that the identity of any individual case could not be ascertained.

### Case definition

Malaria cases are classified as probable or confirmed based on whether they are clinically diagnosed or laboratory confirmed. Clinically diagnosed cases are defined as a patient with malaria-like symptoms who has lived in or recently travelled to areas with known malaria transmission. Laboratory-confirmed cases are defined as clinically diagnosed cases with any positive result from the following laboratory tests related to malaria: malaria parasites confirmed by microscopy, rapid diagnostic tests (RDTs) or polymerase chain reaction test [16]. Physicians in both the public and private medical sectors were required to report malaria cases. Both clinically diagnosed and laboratory-confirmed cases were included in this study.

In China, an imported case of malaria is defined as a malaria case occurring when the patient has travelled to a malaria-endemic country within the previous month. Otherwise, a malaria case is considered to be an indigenous case. Each malaria case was categorized as imported or indigenous by local public health institutes following epidemiological investigation after the case was diagnosed and reported by local physicians [18].

### Study settings

**Fig. 1:**
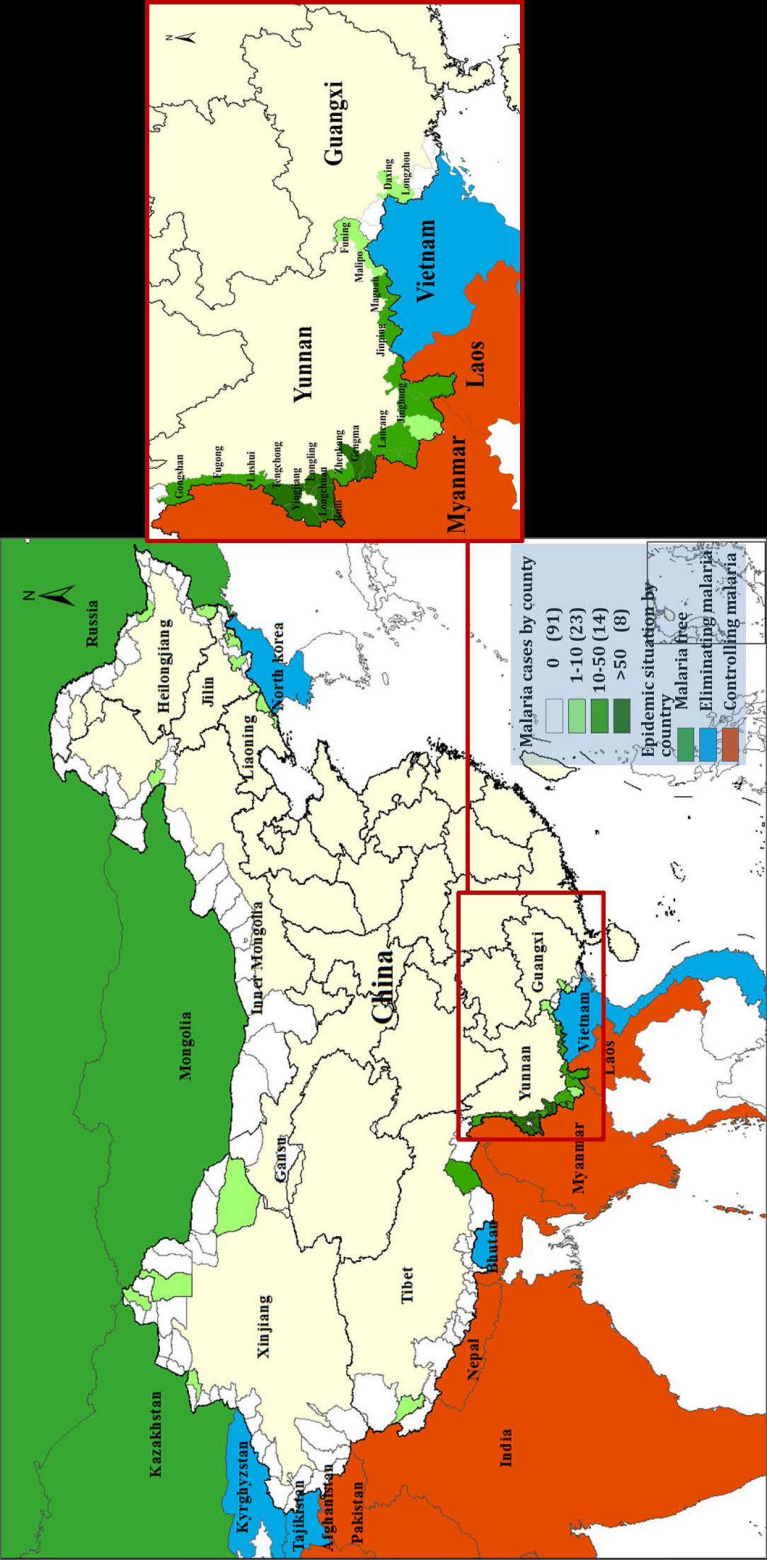
Cumulative malaria cases by county in the land border regions of China, 2011–2014, and the malaria epidemic situation in the adjacent countries in 2012

All 136 Chinese counties with an international land border with any of the 14 neighbouring countries were selected in the current study. These counties are located in nine provinces: Xinjiang, Inner Mongolia, Gansu, Heilongjiang, Jilin, Liaoning, Tibet, Guangxi and Yunnan province [19]. The population density by county ranged from 0.11 to 3017 people/km^2^, with the highest densities mainly concentrated in border counties of Yunnan, Guangxi and Liaoning provinces. Of the 14 adjacent countries, three are malaria free, five have ‘eliminating malaria’ status and six (Myanmar, Laos, India, Nepal, Afghanistan, Pakistan) are still in the ‘controlling malaria stage’ [3, 20] (Fig. 1). Population data for each study county from 2011 to 2014 were retrieved from the National Bureau of Statistics of China. The mean population per county in 2014 was 170,432 people (ranging from 7123 to 639,960).

### Seasonal feature analysis

A seasonal index was used to understand seasonal patterns of malaria incidence. The index for a given month was calculated by the average number of cases for that month during the 4 years of the study, divided by the monthly mean number of cases for all months during the 4 years. No obvious seasonal pattern was expected if the seasonal index of each month was close to 1.0 [21].

### Geographic distribution of disease and spatial analysis

The geographic distribution of cumulative numbers of malaria cases by county during the study period were presented. Furthermore, annual indigenous and imported malaria cases were mapped by county in Yunnan province, separately for each year. The software ArcGIS version 10.2 was used to describe the spatial distribution of malaria using a county-level polygon map.

## Results

### Overall epidemic features

From 2011 to 2014, a total of 1948 malaria cases were reported in 45/136 counties of China sharing international land borders, consisting of 1536 (78.9%) cases of *P. vivax* malaria, 361 (18.5%) cases of *P. falciparum* malaria and 51 (2.6%) other cases, including five (0.25%) cases of *Plasmodium malariae* malaria, three (0.15%) mixed infections and 43 (2.2%) untyped cases. There were 1406 imported cases, 1348 (96%) coming from neighbouring countries and 58 (4%) coming from non-neighbouring countries (Table 1). The proportion of imported cases in *P. vivax* and *P. falciparum* was 78.7 and 21.3%. A total of 542 indigenous cases were identified, occurring in Yunnan (521 cases), Tibet (19 cases) and Liaoning (two cases) provinces. The age distribution showed that the median age of imported cases was 33.0 years and that of indigenous cases was 32.0 years. The cumulative malaria cases by county ranged from 0 to 703. During 2011–14, 88.4% of reported cases were parasitologically confirmed cases and 11.6% were clinically-diagnosed.

**Table 1:** Imported and indigenous malaria cases by province in the land border regions of China, 2011–14

Provinces	No. and length of land bordering counties		Malaria cases				Imported malaria case			Indigenous malaria case		
	No (length)	With malaria occurrence (%)	All	*P. vivax* (%)	*P. falciparum* (%)	Others^a^ (%)	All	From neighbouring country (%)	From non-neighbouring country (%)	All	No. counties in the consecutive three years	
											2011–2013	2012–2014
Yunnan	25 (4060 km)	25 (100)	1878	1520 (80.9)	324 (17.3)	34 (1.8)	1357	1342 (98.9)	15 (1.1)	521	11	7
Liaoning	5 (128 km)	5 (100)	27	3 (11.1)	22 (81.5)	2 (7.4)	25	1 (4.0)	24 (96.0)	2	0	0
Tibet	18 (3842 km)	2 (11.1)	20	10 (50.0)	0	10 (50.0)	1	0	1 (100)	19	1	1
Jilin	10 (1438 km)	4 (40.0)	9	0	7 (77.8)	2 (22.2)	9	0	9 (100)	0	0	0
Guangxi	8 (800 km)	2 (25.0)	7	2 (28.6)	3 (42.9)	2 (28.6)	7	5 (71.4)	2 (28.6)	0	0	0
Xinjiang	32 (5600 km)	4 (12.5)	4	0	3 (75.0)	1 (25.0)	4	0	4 (100)	0	0	0
Heilongjiang	18 (3045 km)	2 (11.1)	2	1 (50.0)	1 (50.0)	0	2	0	2 (100)	0	0	0
Inner Mongolia	19 (4200 km)	1 (5.3)	1	0	1 (100)	0	1	0	1 (100)	0	0	0
Gansu	1 (65 km)	0	0	0	0	0	0	0	0	0	0	0
Overall	136	45 (33.1)	1948	1536 (78.9)	361 (18.5)	51 (2.6)	1406	1348 (95.9)	58 (4.1)	542	12	8

^a^Others contained *P. malariae*, mixed infection cases and untyped cases

In the three consecutive years from 2012 to 2014 and in the four consecutive years from 2011 to 2014, indigenous malaria cases continued to occur in seven counties of Yunnan province and in Motuo county of the Tibet autonomous region.

Among the counties in the north and west of China, most had no malaria cases, with only a small number of sporadic cases. By contrast, in the counties of the south of China, there were eight counties with more than 50 cumulative cases, all of which were located in Yunnan province and adjacent to Myanmar, which is at the malaria control stage (Fig. 1).

**Fig. 2:**
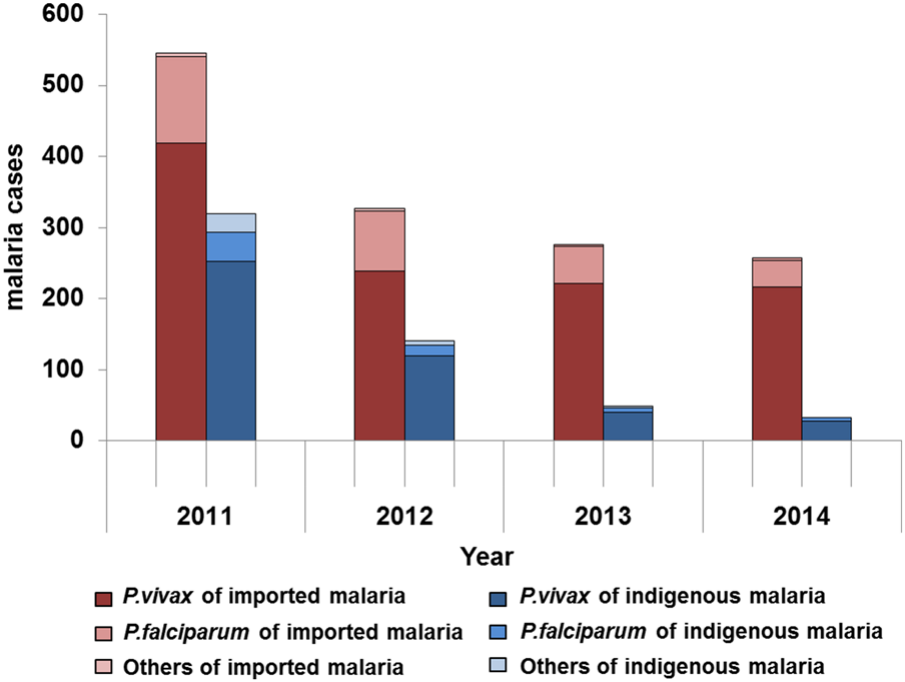
The cases of imported and indigenous malaria in the land border areas of China, 2011–2014

Imported malaria cases were reported each year from 2011 to 2014, with a peak of 546 cases in 2011, decreasing yearly in the following years. The proportion of imported cases in *P. vivax* and *P. falciparum* was 78.7 and 21.3%. The number of imported cases caused by *P. vivax* decreased by 43.0% in 2012 (from 419 cases in 2011 to 239 cases in 2012) and was stable during the following years (216 cases in 2014). Imported cases caused by *P. falciparum* decreased from 121 cases in 2011 to 38 cases in 2014 (Fig. 2). The number of counties with imported cases slightly decreased from 28 counties in 2011 to 26 counties in 2014. The number of counties affected by *P. vivax* decreased from 21 in 2011 to 17 in 2014, and the number affected by *P. falciparum* decreased from 18 in 2011 to 15 in 2014.

**Fig. 3:**
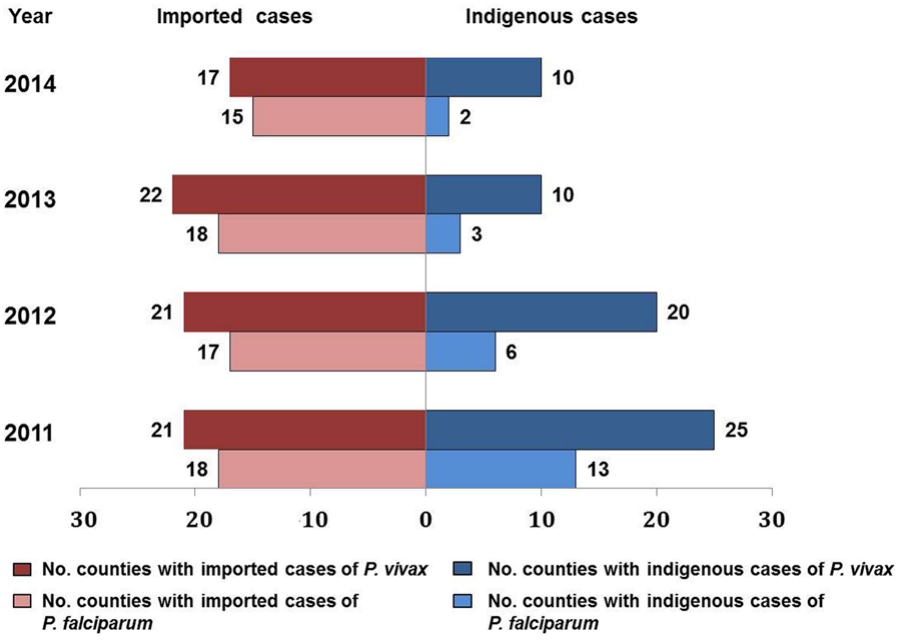
Change in the number of counties affected by imported and indigenous malaria by year in the land border areas of China, 2011–2014

Indigenous malaria cases presented a markedly decreasing trend during the period from 2011 to 2014, with 319 indigenous cases in 2011 reducing to only 33 indigenous cases in 2014. Indigenous cases caused by *P. vivax* decreased from 253 cases in 2011 to 28 cases in 2014, and indigenous cases caused by *P. falciparum* decreased from 40 cases in 2011 to five cases in 2014. The number of counties with indigenous cases reduced from 26 counties in 2011 to 10 counties in 2014. The number of counties with indigenous cases of by *P. vivax* decreased from 25 in 2011 to 10 in 2014, and the number of counties affected by *P. falciparum* decreased from 13 in 2011 to two in 2014 (Fig. 3).

### Seasonality

**Fig. 4:**
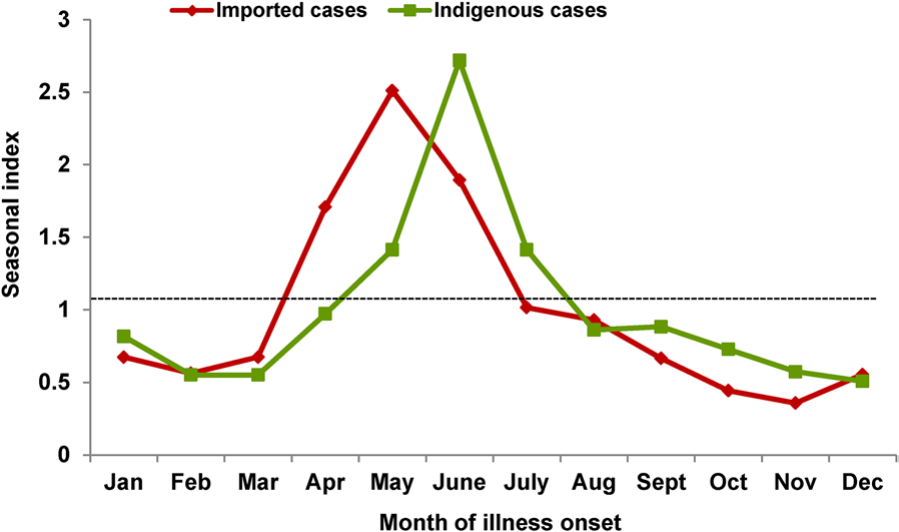
The seasonal index of imported cases and indigenous cases in the land border areas of China, 2011–2014

Malaria cases in the border counties presented obvious seasonal characteristics during the study period. In these counties, 51.0% of imported cases were reported between April and June, with a peak in May. The seasonal index was the highest (2.5) in May. Indigenous cases occurred most frequently between May and July, when 46.2% of cases were reported. The peak of indigenous case occurrence was in June, and the seasonal index was 2.7, with a lag of 1 month from that of imported cases (Fig. 4).

### The border areas in Yunnan province

**Fig. 5:**
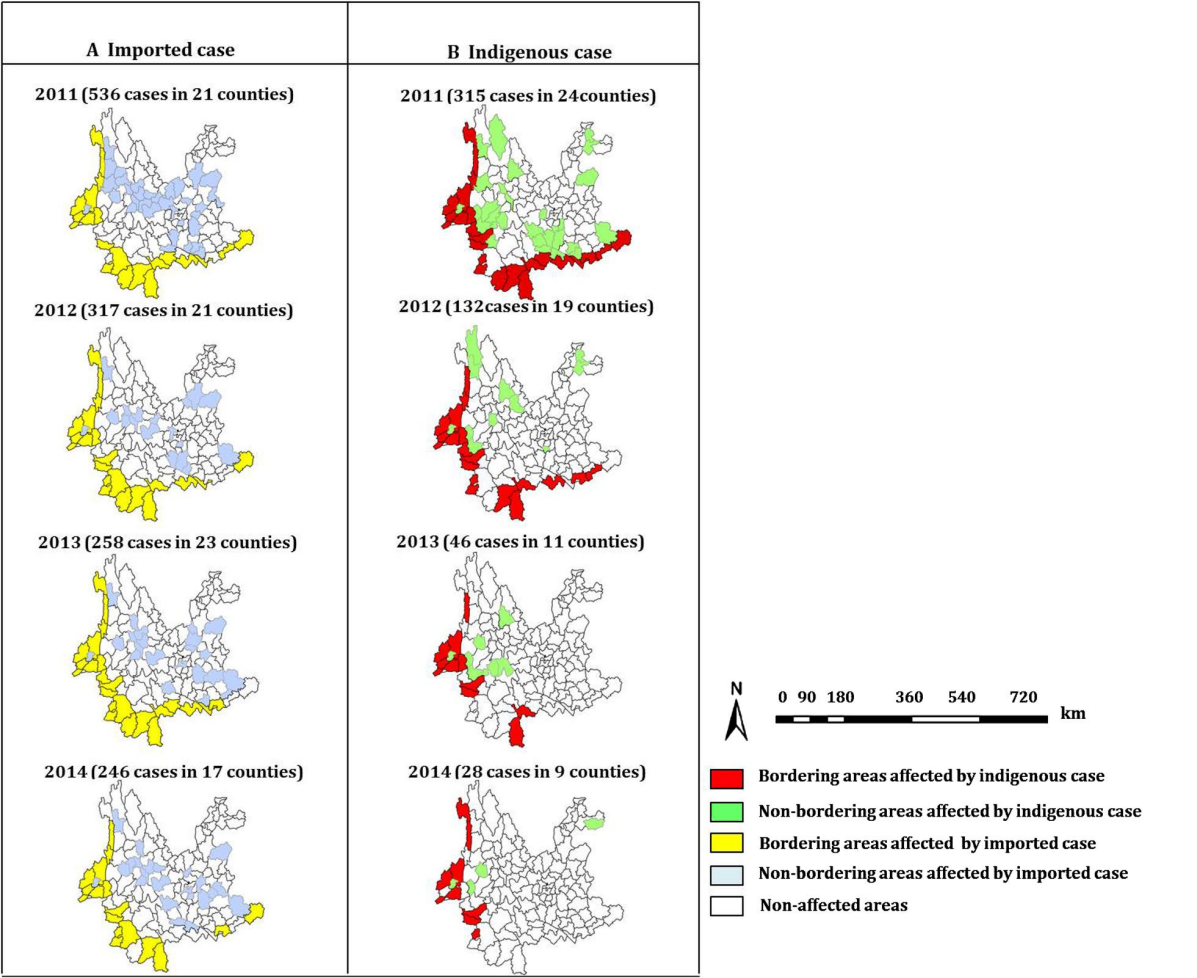
The change in counties affected by imported and indigenous malaria in the international border counties of Yunnan province by year, 2011–2014

Of the total malaria cases from 2011 to 2014, 96% (1878 cases) occurred in Yunnan province. Yunnan has 25 land border counties adjacent to Myanmar, Vietnam and Laos. From 2011 to 2014, 1878 cases were reported, of which 1357 were imported and 521 were indigenous. 98.9% (1342 cases) of imported cases came from neighbouring countries (95.9% from Myanmar, 4.1% from Laos), and 1.1% (15 cases) came from non-neighbouring countries (African countries, Cambodia and Thailand). The number of counties with imported cases decreased from 21 counties in 2011 to 17 counties in 2014. Indigenous malaria cases decreased substantially from 315 cases in 2011 to 28 cases in 2014. The counties with indigenous cases also decreased substantially from 24 counties in 2011 to 9 counties in 2014. In the border counties adjacent to Laos and Vietnam, malaria cases declined markedly from 73 cases in 2011 to seven cases in 2014. Indigenous cases were not reported in these counties in 2014, and there were only a few imported cases. All seven bordering counties of Yunnan province where indigenous malaria cases occurred constantly in the three consecutive years from 2012 to 2014 were adjacent to Myanmar (Fig. 5).

## Discussion

This study found that the magnitude and geographic distribution of malaria in the international land border counties of China decreased dramatically since the initiation of the malaria elimination programme in 2010. There has been a lot more imported malaria than indigenous malaria in these regions.

Regional diversity affects the epidemiological characteristics of malaria in the land border regions in northern, western and southern China. In northern and western China, including Inner Mongolia, Xinjiang, Gansu, Heilongjiang and Jilin provinces, low population density and climate are not conducive to the transmission of malaria. Therefore, few local malaria cases were confirmed in these provinces. Moreover, the neighbouring countries of these provinces are generally low-transmission settings. The WHO categorizes Mongolia, Russia and Kazakhstan as malaria-free countries, and they present almost no risk of introducing malaria into China [22].

Nevertheless, in Liaoning province, indigenous malaria recently emerged in Dandong city. Dandong city is close to the border with North Korea, with frequent movement of people and goods across the border, and mosquito vectors capable of malaria transmission [23–26]. Currently, the risk of malaria spreading from North Korea to China is hard to estimate, because the epidemic situation in North Korea is uncertain [3].

In Motuo county of Tibet, the local humid and sub-tropical monsoon climate is suitable for mosquitoes breeding. Because it is a remote area, healthcare services have difficulty reaching the local population [27]. In order to achieve the nationwide elimination goal, malaria prevention and treatment should be prioritized in such hard-to-reach areas.

There were most reported malaria cases in Yunnan province, and the adjacent countries of Yunnan were in the controlling malaria stage. Hence, Yunnan province was further focused on. Several counties in Yunnan with persistent indigenous malaria cases, especially along the border with Myanmar, pose the greatest threat to achieving national malaria elimination [28]. However, it is encouraging that malaria in the land border regions of Yunnan province was effectively controlled in recent years by joint and cross-border prevention and control strategies. Indigenous transmission of malaria has been interrupted in many regions. The strategies used include timely detection, diagnosis and appropriate treatment for malaria cases; finding the source of infection promptly in all land border counties; setting up malaria prevention stations in the border points; and active screening in exported labour to reduce the spread of malaria out of China [29–31].

Nevertheless, indigenous malaria cases still consistently occurred in several land border counties during this study. The border of Yunnan province is nearly 4060 km, with 18 border ports and 643 border pathways. Because Yunnan province has the highest border region population density, frequent cross-border travel and difficulty managing the migrant population, prevention and control of malaria a more complex [32, 33]. The key Challenges of elimination by 2020 are mainly related to cross-border and imported malaria. Therefore, more sensitive and quicker responses for case identification are necessary. Village-level capacity for malaria diagnosis, treatment and county-level surveillance and management of exported labour should be strengthened to prevent local secondary malaria case occurrence [34]. International collaborations as the Asia Pacific Malaria Elimination Network, the Asia Pacific Leaders Malaria Alliance and China’s Belt and Road Initiative need to be further enhanced for the control of imported malaria.

This study only focused on malaria risk in land border regions, because China has long land borders and some neighbouring countries are highly-endemic for malaria. However, in today’s world, malaria can be imported through air and sea transportation, and this could also potentially reintroduce local transmission in areas in the interior with conditions conducive to malaria transmission. For example, a large outbreak of imported malaria occurred in 2013 in Shanglin county, the inner county of Guangxi autonomous regions, which arose from Chinese gold miners returning from overseas (30). Further study of malaria importation and reintroduction should occur in regions with concentrated air and sea transportation and high levels of tourism.

## Conclusions

The scale and extent of malaria occurrence in the international border counties of China have decreased sharply during implementation of the national malaria elimination programme. However, areas along the China–Myanmar border are still facing a high risk of malaria introduction and local transmission. Malaria infection in these hotspots is the main threat to achieving and maintaining malaria elimination in China. Surveillance and elimination strategies should be adjusted to account for these changes, and further research should explore the features of these areas to achieve the goal of national malaria elimination by 2020.
